# How machine-learning recommendations influence clinician treatment selections: the example of antidepressant selection

**DOI:** 10.1038/s41398-021-01224-x

**Published:** 2021-02-04

**Authors:** Maia Jacobs, Melanie F. Pradier, Thomas H. McCoy, Roy H. Perlis, Finale Doshi-Velez, Krzysztof Z. Gajos

**Affiliations:** 1 Department of Computer Science, Harvard University, 29 Oxford Street, Cambridge, MA 02138 USA; 2 Center for Quantitative Health, Massachusetts General Hospital, 185 Cambridge Street, Boston, MA 02114 USA; 3 Harvard Medical School, 25 Shattuck Street, Boston, MA 02115 USA

**Keywords:** Depression, Scientific community

## Abstract

Decision support systems embodying machine learning models offer the promise of an improved standard of care for major depressive disorder, but little is known about how clinicians’ treatment decisions will be influenced by machine learning recommendations and explanations. We used a within-subject factorial experiment to present 220 clinicians with patient vignettes, each with or without a machine-learning (ML) recommendation and one of the multiple forms of explanation. We found that interacting with ML recommendations did not significantly improve clinicians’ treatment selection accuracy, assessed as concordance with expert psychopharmacologist consensus, compared to baseline scenarios in which clinicians made treatment decisions independently. Interacting with *incorrect* recommendations paired with explanations that included limited but easily interpretable information did lead to a significant reduction in treatment selection accuracy compared to baseline questions. These results suggest that incorrect ML recommendations may adversely impact clinician treatment selections and that explanations are insufficient for addressing overreliance on imperfect ML algorithms. More generally, our findings challenge the common assumption that clinicians interacting with ML tools will perform better than either clinicians or ML algorithms individually.

## Introduction

Researchers are rapidly demonstrating the potential benefits of predictive analytics across mental health research, including neuroimaging^1^, behavioral modeling^2^, and pharmacotherapy^3^. However, despite the proliferation of machine-learning (ML) models for healthcare, these tools have not yet meaningfully influenced real-world clinical care^4–7^. A significant barrier to implementation is a lack of research assessing how ML recommendations may be used by clinicians and influence their decision-making processes. Researchers have therefore called for model integration techniques that meaningfully engage with clinical stakeholders to understand real-world interpretability and utility^5,8^.

Toward the goal of translating ML to real-world decision support tools, we explore to what extent clinical practice could be improved if clinicians were presented with recommendations produced by such models. Our work is motivated by a vast literature on intelligent decision support systems, which for example includes the design of cockpits and criminal risk assessments, which suggests that the way in which information is presented to decisionmakers can have a significant impact on their performance^9–13^. Two key elements that emerge from this literature are the negative impact of incorrect information on performance (i.e., if a model recommends a wrong choice), and the ability for explanations about why a recommendation is made to encourage reliance on the recommendation itself. More work is needed to understand how these elements will influence clinical behaviors.

To address this gap, we conducted an experiment with 220 clinical-care providers to assess the impact of ML treatment recommendations on clinician treatment selection. The possibility of improving treatment outcomes in major depressive disorder (MDD) using ML has received increased attention in recent years^14–17^. Identifying optimal treatment in this context is particularly challenging because of heterogeneous symptoms, tolerability concerns, and the prevalence of treatment-resistant depression, which can result in clinicians and patients using trial and error to find an effective treatment^18,19^. This process can be inefficient, with one-third of patients failing to reach remission after up to four antidepressant trials^18,20^. Multiple ML models have been proposed to aid in MDD treatment selection^21–24^.

We evaluated two research questions: (1) How do correct and incorrect ML recommendations influence clinicians’ antidepressant selection accuracy, decision confidence, and perceived utility of the recommendation? (2) How do different types of supporting explanations for the recommendation influence treatment selection accuracy, decision confidence, and perceived utility of the recommendation? Our results highlight the importance of evaluating clinician-model collaborative behaviors and clinician responses to ML model errors.

## Materials and methods

### Experimental design

We applied a within-subject factorial study design using patient vignettes. In the experiment, participants were presented with a series of questions, where each question included a random vignette paired with a treatment recommendation and a form of explanation for that recommendation. These independent variables were systematically varied with each question so that participants saw all combinations. This method provides an efficient means of investigating the influence of multiple independent variables on multiple dependent variables^25^ and has been useful for assessing judgments related to complex beliefs and behaviors^26,27^. We elaborate on the variables included in this study below.

#### Independent variables

*Recommendation concordance with expert consensus*. Conditions included no recommendation (baseline), correct, or incorrect. Correct and incorrect recommendations for each patient vignette were determined by five experts in psychopharmacology, as described in the “Treatment selection accuracy” section. Correct recommendations included the top-scored antidepressant across the five psychopharmacologists (all with a mode score of 1, denoting the best choice). Incorrect recommendations included the lowest-scored antidepressant (all with a mode score of 0, denoting a poor choice). While we highlighted a single recommendation for each vignette, we also showed a top-5 list of recommended treatment options, recognizing that there are often several reasonable options in a given context. Informed by recent work using medical record data to predict treatment success^24,28^, we presented treatment options with associated stability scores and dropout risk scores. Stability is defined as the predicted likelihood that a patient will continue to use the associated treatment for at least 3 months. Dropout is defined as the risk that the patient will discontinue the associated treatment. For consistency, the top-recommended treatment was associated with a stability score between 0.70 and 0.80 and a dropout risk score between 0.01 and 0.11.

**Fig. 1:**
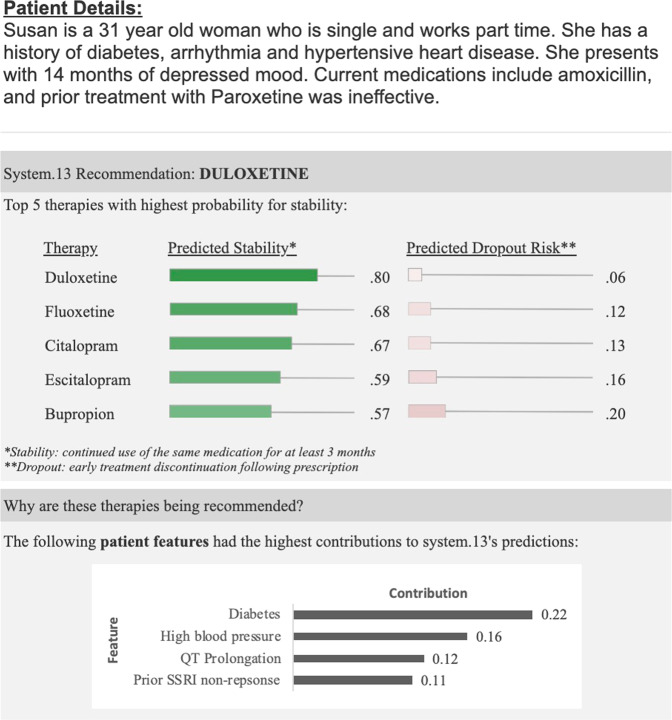
A sample study question. Each question includes a random patient vignette and may include a recommendation and an explanation. This example shows a patient vignette with a correct recommendation and a feature-important explanation.

*Explanation type*. Conditions included none, placebo, feature-based, heuristic-based. With no explanation, a participant was only presented with the treatment recommendations. Placebo’s explanations stated that “recommendations are based on patients’ ICD-9 codes”. We included placebo explanations to distinguish between effects caused by the visibility of an explanation and the *content* of an explanation. Clinical feature-based explanations highlighted four aspects of a patients’ medical history that contributed to the machine learning prediction, as shown in Fig. 1. Finally, we included a heuristic-based explanation, which showed relevant prescription heuristics for relevant aspects of a patients’ history. These heuristics were curated by two expert academic psychopharmacologists with a mean of 12 years in clinical practice and they included indications and contraindications based on a patient’s symptoms or comorbidities. An example of such a heuristic would be favoring sedating drugs for patients experiencing insomnia. See Supplementary Table 1 for the full list of heuristics used in this study. Both the feature-based and heuristic-based explanations were selected as these approaches had previously been investigated in other domains^29^.

#### Dependent variables

*Treatment selection accuracy*. To determine accuracy scores, five experienced academic psychopharmacologists with a mean of 26 years in clinical practice scored 24 antidepressant treatment options for each patient vignette. They used a 3-point rating scale: 0 = worst choice, 0.5 = fair choice, 1 = best choice. We used the mode of their ratings to assign a value for each antidepressant for each vignette. For each vignette, the participant was assigned that value based on the antidepressant they selected. We removed from analysis any treatment selections not included in the 24 antidepressants scored by the psychopharmacologists. Supplementary Table 2 shows the group sizes included in the analysis.

*Treatment selection confidence*. In each vignette, after selecting a treatment, participants were asked “How confident are you with this decision?” using a 5-point Likert scale (1 = not at all confident, 5 = extremely confident).

*Perceived utility*. For each vignette, participants were asked to rate how helpful the ML recommendation was in making their decision, using a 5-point Likert scale (1 = not at all, 5 = a great deal).

#### Random variable

**Fig. 2:**
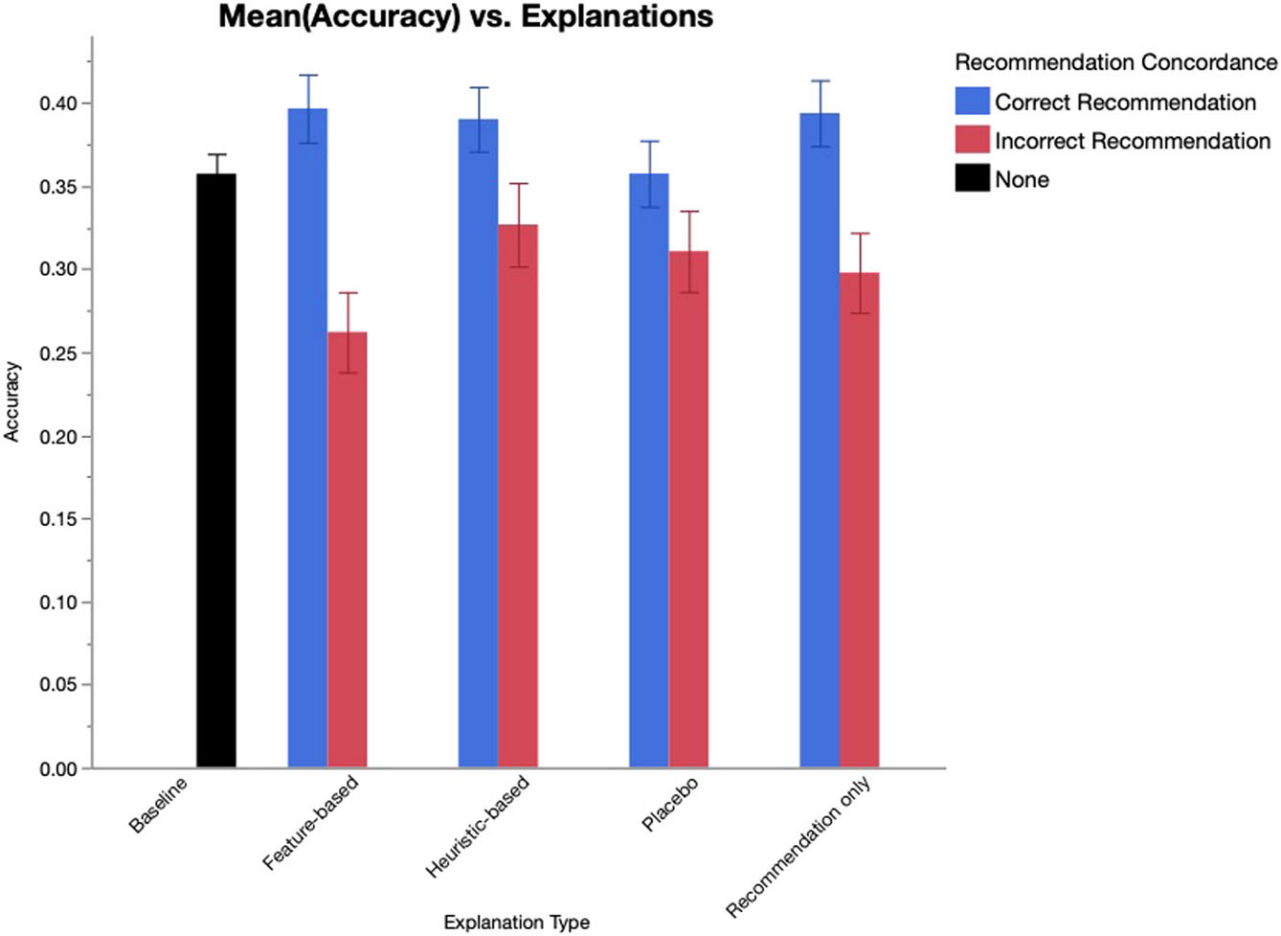
Changes in mean treatment selection accuracy by explanation type for the baseline, correct, and incorrect recommendation conditions. Error bars show standard error.

*Patient vignette*. We created five hypothetical patient descriptions. Each description included a patient’s name, age, and employment status (as a distractor). The description also included the length of time since depression symptoms began, one contraindication, one irrelevant contraindication, and one previously ineffective selective serotonin reuptake inhibitor trial. For each question, one of the five patient vignettes were randomly displayed. Due to the repeated use of vignettes, the patients’ names and ages were changed each time the vignette was displayed. To reduce the risk of confounding variables, we limited the age range to 33–43 years. The full set of vignettes are listed in Supplementary Table 1. Figure 2 shows a complete sample question, including a patient vignette, machine learning recommendation, and explanation. As shown in the figure, participants have presented the top 5 options based on model output, all of which were considered reasonable options by expert consensus. The varying scores represent the algorithm confidence scores. For the incorrect condition, only the top recommendation was changed. In the example vignette shown in Fig. 2, for the incorrect condition, Venlafaxine was included in the list. In light of the patient’s history of hypertensive heart disease, Venlafaxine is considered a less-preferred treatment option for the patient described in the vignette, consistent with published guidelines^30,31^.

### Task description

We showed participants a total of 17 scenarios. This included five baseline scenarios, in which each patient vignette was displayed once without any ML recommendation. The 12 other scenarios included a patient vignette, a recommendation, and an explanation. The recommendations did not come directly from an ML algorithm. Instead, the ML outputs were simulated and were manipulated as part of the experiment to assess the risks of algorithmic errors. We included one incorrect recommendation for every two correct recommendations. To reduce the risk of participants creating general assumptions about the model accuracy, we labeled each recommendation as being derived from a different ML model. Each participant saw the scenarios in random order. For each scenario, participants were asked to make an antidepressant treatment selection for the patient, rate their decision confidence, and indicate to what extent the ML recommendation helped them to make their decision.

### Procedures

The study received ethical approval from the Harvard University Institutional Review Board (protocol: IRB18-1603). The study was conducted online using the Qualtrics XM Platform. We recruited participants using social media and snowball sampling between August 17–18, 2019, including an advertisement on a continuing medical education Facebook page. Participants first provided online informed consent. Participants then completed a demographics questionnaire, followed by the experimental task. We provided each participant with a $20 gift card.

### Statistical analysis

Per protocol, we used a repeated-measures ANOVA for within-subjects analyses and a two-sided Student’s *t* test for post hoc independent pairwise comparisons. We controlled for the patient vignette, display order, and participants’ medical specialty, age, years of medical experience, and ML familiarity. To address the problem of multiple comparisons, we adopted a Bonferroni correction for all post hoc pairwise analyses, with an alpha value of 0.05. We include the adjusted *p* value for all post hoc pairwise comparisons, and report effect sizes using Cohen’s *d*. Data were checked for normality (Shapiro–Wilk *W* = 0.992, *p* = 0.287). Power exceeds 80% to detect an effect size (Cohen’s *d*) of 0.2 or greater with 200 participants and alpha = 0.05. All data analyses were conducted using JMP Pro v14.

## Results

In total, 240 clinicians completed the web-based experiment, of which 20 were removed due to ineligibility, leaving 220 for analysis. Table 1 shows descriptive statistics of the 220 participants included in the analysis. Reasons for ineligibility included having <1 year of experience prescribing antidepressant treatments [6; 2.5%] or not providing a medical specialty [3; 1.25%]. We also removed responses from outside of the United States [12; 5.0%] due to the small response rate and possible differences in training and treatment selection processes.

**Table 1:** Descriptive statistics of the 220 study participants, including age, medical specialty, and years of experience prescribing antidepressants, and machine-learning familiarity.

Age (SD)	42.52 (9.28)
*Medical specialty (%)*	
Psychiatry	195 (88.64)
Primary Care	18 (8.18)
Other	7 (3.18)
Years of experience prescribing antidepressants (IQR)	10 (7–15)
*Machine-learning familiarity (%)*	
Extremely familiar	45 (20.45)
Very familiar	51 (23.18)
Moderately familiar	30 (13.64)
Slightly familiar	54 (24.55)
Not familiar at all	40 (18.18)

### No change in overall accuracy between clinicians and clinician-ML collaboration

We first compared the overall performance of clinicians’ baseline decisions (with no ML recommendations), the simulated ML system, and the clinician–ML collaborative performance. Accuracy scores could range from 0 to 1, with 1 corresponding to making the optimal choice for every vignette based on expert psychopharmacologist consensus. The simulated ML system, if acting independently, was designed to have an overall accuracy score of 0.667, calculated as the accuracy scores of the top-recommended treatment for each question that included an ML recommendation. This accuracy score was significantly greater than clinicians’ mean score of 0.357 (95% CI: 0.333–0.381; *t*(3531) = −19.21; *p* < 0.0001, *d* = 0.74), which we calculated by averaging participants’ scores across the baseline scenarios, which did not include an ML recommendation. The ML accuracy score was also significantly greater than the clinician–ML collaborative performance (*M* = 0.356, 95% CI: 0.340–0.371; *t*(4758) = −25.63; *p* < 0.0001, *d* = 0.75), which we calculated using the mean accuracy scores of participants’ responses to the scenarios that included an ML prediction. We did not observe a significant difference between clinician performance when acting independently compared with the clinician–ML collaborative performance (*t*(3011) = 0.094; *p* = 0.925).

### Incorrect recommendations significantly lowered treatment selection accuracy

We observed a main effect of recommendation concordance with experts on treatment selection accuracy (*F*_2,2697_ = 13.41, *p* < 0.0001). Table 2 summarizes the results of this analysis. Post hoc pairwise comparisons with Bonferroni correction showed that incorrect recommendations correlated with significantly lower accuracy scores (*M* = 0.299, 95% CI: 0.275–0.322) compared to correct recommendations (*M* = 0.384, 95% CI: 0.365–0.403; *t*(2118) = −5.19; *p* < 0.0001, *d* = 0.24), and compared to baseline conditions with no recommendation (*M* = 0.357, 95% CI: 0.334–0.381; *t*(1601) = 3.44; *p* = 0.0018, *d* = 0.16). We observed no significant difference in scores between correct recommendations and baseline conditions (*t*(2301) = −1.54, *p* = 0.366).

**Table 2:** Accuracy, confidence, and utility scores stratified by recommendation correctness.

Recommendation correctness	Accuracy		Confidence		Perceived utility	
	*M* (95% CI)		*M* (95% CI)		*M* (95% CI)	
Baseline	0.357 (0.333–0.381)	*p* < 0.0001*	3.67 (3.63–3.72)	*p* = 0.133	N/A	*p* = 0.0006*
Correct	0.384 (0.365–0.403)		3.65 (3.62–3.69)		3.52 (3.47–3.56)	
Incorrect	0.299 (0.275–0.322)		3.62 (3.57–3.69)		3.40 (3.32–3.47)	

*p*-values measured using repeated-measures ANOVA with a significance level of 0.05.

We also observed a main effect of recommendation concordance on the perceived utility of the ML system (*F*_1,2315_ = 11.72, *p* = 0.0006). Correct recommendations correlated with significantly higher utility scores (*M* = 3.52, 95% CI: 3.47–3.56) compared to incorrect recommendations (*M* = 3.40, 95% CI: 3.32–3.47; *t*(2629) = −3.42; *p* = 0.0012, *d* = 0.11).

We did not observe a main effect of recommendation concordance on clinicians’ treatment selection confidence between baseline conditions (*M* = 3.67 95% CI: 3.63–3.72), correct recommendations (*M* = 3.65, 95% CI: 3.62–3.69), or incorrect recommendations (*M* = 3.62 95% CI: 3.57–3.67; *F*_2,3379_ = 2.02, *p* = 0.133).

### Influence of explanations on performance metrics

To assess the impact of explanations on performance, we first examined the effects of explanation type when paired with correct recommendations, followed by effects of explanation type when paired with incorrect recommendations. The results are summarized in Table 3.

**Table 3:** Accuracy, confidence, and utility scores stratified by explanation type for correct and incorrect recommendations.

Recommendation correctness	Explanation type	Accuracy		Confidence		Perceived utility	
Correct recommendations	Baseline	0.357 (0.333–0.381)	*p* = 0.239	3.67 (3.63–3.72)	*p* = 0.239	N/A	*p* = 0.343
	No explanation	0.394 (0.355–0.433)		3.64 (3.57–3.72)		3.45 (3.35–3.55)	
	Placebo	0.357 (0.318–0.397)		3.66 (3.59–3.74)		3.53 (3.43–3.63)	
	Feature based	0.397 (0.356–0.437)		3.62 (3.54–3.69)		3.54 (3.44–3.64)	
	Heuristic based	0.390 (0.352–0.428)		3.70 (3.62–3.77)		3.54 (3.44–3.64)	
Incorrect recommendations	Baseline	0.357 (0.333–0.381)	*p* = 0.004*	3.67 (3.63–3.72)	*p* = 0.155	N/A	*p* = 0.573
	No explanation	0.298 (0.250–0.345)		3.60 (3.50–3.70)		3.38 (3.23–3.53)	
	Placebo	0.311 (0.262–0.359)		3.65 (3.54–3.76)		3.36 (3.21–3.51)	
	Feature based	0.262 (0.214–0.310)		3.67 (3.56–3.77)		3.48 (3.34–3.63)	
	Heuristic based	0.327 (0.277–0.376)		3.57 (3.46–3.68)		3.36 (3.20–3.52)	

*p*-values measured using repeated-measures ANOVA with a significance level of 0.05.

#### No significant effects on dependent variables when explanations are paired with correct recommendation

When clinicians were presented with no recommendations (baseline) or correct recommendations, we observed no effect of explanation type on treatment selection accuracy (*F*_4,2017_ = 1.38, *p* = 0.239), treatment selection confidence (*F*_4,2526_ = 1.38, *p* = 0.239), or perceived ML utility (*F*_3,1465_ = 1.11, *p* = 0.343).

#### Feature-based explanations lower treatment selection accuracy when paired with incorrect recommendations

When clinicians were presented with no recommendations or incorrect recommendations, we observed a main effect of explanation type on accuracy (*F*_4,1350_ = 3.86, *p* = 0.004). Post hoc pairwise comparisons with Bonferroni correction showed that incorrect recommendations paired with feature-based explanations correlated with significantly lower accuracy scores (*M* = 0.262, 95% CI: 0.214–0.310) compared to the baseline condition (*M* = 0.357, 95% CI: 0.333–0.381; *t*(1426) = −3.57; *p* = 0.004, *d* = 0.28). Figure 2 shows the difference in treatment selection accuracy across the explanation types for the baseline, correct, and incorrect recommendation conditions. We did not observe a significant difference in accuracy scores across the other explanation types. We provide all post hoc pairwise comparisons in Supplementary Table 3. We also did not observe any effect of explanation type on treatment selection confidence (*F*_4,1678_ = 1.67, *p* = 0.155) or on perceived ML utility (*F*_3,612.3_ = 0.667, *p* = 0.573).

### Influence of ML familiarity on performance metrics

We measured ML familiarity using a 5-point Likert scale (1 = Not at all familiar, 5 = Extremely familiar). We found that clinicians who rated their familiarity with ML as “Not at all familiar” were seven times more likely to select a treatment that aligned with the ML recommendation (34% concordance) compared to clinicians who said they were “extremely familiar” with ML (5% concordance). We used a Pearson correlation to examine the association between ML familiarity and the dependent variables. We observed a statistically significant association between ML familiarity and treatment selection accuracy (*r* = −0.188, *p* < 0.0001), suggesting that with increased ML familiarity, treatment selection accuracy decreased. We also observed a statistically significant correlation between ML familiarity and confidence (*r* = 0.129, *p* < 0.0001), indicating that with increased ML familiarity, treatment selection confidence increased. Finally, we observed a significant association between ML familiarity and perceived utility of the ML recommendation (*r* = 0.317, *p* < 0.0001), suggesting that as ML familiarity increased, perceived ML utility also increased, despite the reduced use of the ML recommendation.

## Discussion

In this study of 220 antidepressant prescribing clinicians, we found that interacting with ML recommendations did not improve treatment selection accuracy, where accuracy was assessed based on concordance with psychopharmacology experts. In this experiment, the ML results were simulated and manipulated as part of the experiment to assess how clinicians respond to algorithmic errors. Interacting with incorrect recommendations did correlate with significantly lower treatment selection accuracy scores compared to correct recommendations and questions with no ML recommendation, though clinicians also scored the ML recommendations as less helpful when the recommendation was incorrect. We also observed an effect of explanation type on treatment selection accuracy. When paired with incorrect recommendations, interacting with feature-based explanations correlated with lower accuracy scores compared to the baseline condition. Through this study, we demonstrate the potential risks of ML applications and demonstrate how ML errors may negatively influence clinical decisions. Our results show the importance of human factors research and methods in designing ML for clinical decision-making.

Several recent studies have evaluated ML models with the intended use of creating decision support systems^32,33^, many of which focused on psychiatric care^2,22,24,28^. One assumption from the broader ML community that motivates this research is that humans interacting with ML tools will perform better than either actor individually^34,35^. Recent perspectives have also discussed the potential for ML tools to influence health care decisions and outcomes^36–38^. However, we found few studies that evaluate how medical experts change their behaviors when interacting with diagnostic recommendations. In an ideal setting, a clinician supported by an ML predictor would make fewer errors than a clinician or predictor alone. Our work challenges the validity of this assumption, aligning with recent studies in non-medical domains suggesting that humans interacting with ML tools may perform worse than the algorithm acting independently^39,40^.

Explanations are a common approach for encouraging appropriate trust in ML tools. Studies have suggested that ML explanations may increase trust in the technology^39,41^, but in some cases, this can lead to an overreliance on the algorithms^42^. Our study helps to unpack the complicated influence of explanations on behavior, demonstrating how different explanation types influence clinicians’ treatment selection. We identified a significant reduction in accuracy scores when comparing baseline conditions (no ML recommendation) to feature-based explanations with incorrect recommendations, indicating that explanations did not effectively address accuracy issues caused by incorrect recommendations and can exacerbate issues of overreliance. Prior research found that simple explanations, where simplicity was defined as the number of causes, were considered more probable by participants^43^. While this experiment did not differ on the number of causes included in the explanation, feature-based explanations included more limited information compared to heuristic-based explanations, which may help to explain the increased use of these explanations and reduced accuracy scores. While there is interest in developing clinical-facing technologies that are visually simple, our results suggest that less information within an explanation is not always better. The effect of explanation type on accuracy scores demonstrates that in addition to commonly discussed issues of technical readiness and data bias^6^, the interface design of ML decision support tools must be systematically evaluated. Design decisions, such as the type of explanation to display, can have significant effects on clinicians’ behavior. Future work needs to continue to consider the trade-off between effectiveness and usability in order to optimize for clinician–ML collaboration.

Importantly, we found that the subjective metrics (confidence and ML utility) followed different trends compared to the metric of treatment selection accuracy. We identified no significant change in confidence between the baseline conditions, correct recommendations, or incorrect recommendations. The lack of change in confidence scores suggests that participants struggled to calibrate their own performance, which can lead to poor calibration of ML performance^11^. While utility scores were lower for incorrect recommendations, we found no effect of explanation type on perceived utility, despite explanation type having an effect on accuracy. These findings align with a small but growing body of work suggesting that subjective measures cannot be used to predict the success of decision support tools^40,44,45^.

Finally, we found in secondary analyses that clinicians with higher familiarity with ML were less likely to use an ML recommendation compared to clinicians with lower ML familiarity. Our results point to a need for future research to consider how and why clinicians’ experience with ML may influence their engagement and trust in ML systems.

Our results may have important implications as ML tools become increasingly prevalent in psychiatry workflows. While clinicians’ acceptance of the technology and the performance of the algorithms are both crucial to adoption, our results indicate that these factors are not enough to be able to predict positive performance outcomes. Evaluation techniques using realistic tasks and settings with the target user are necessary for determining how ML recommendations influence clinical decisions. As such models are examined, our results also suggest the importance of considering the impact of incorrect recommendations, and the extent to which explanation methods may reduce the negative impact of such recommendations. Finally, our results suggest that prior experience with ML models may influence clinicians’ willingness to use ML recommendations in treatment selection decisions. Therefore, strategies for developing appropriate levels of trust with ML models ought to account for, and be tailored to, clinicians’ familiarity with ML.

We note multiple limitations in these experiments. First, we did not include medication combinations or nonpharmacological treatments, and the extent to which our results generalize to these contexts will require further study. Second, although we focused on how recommendation concordance and explanation style influenced participant responses, other aspects of the ML recommendation merit consideration. For example, communicating the confidence level of the recommendation may influence the use of the recommendation itself. Additionally, the study was deployed as an online behavioral experiment using hypothetical patient scenarios. Next-step studies should also examine these models in real-world clinical workflows. Finally, all psychopharmacology experts involved in this study came from the northeast United States. While these psychopharmacologists taught both national and international CME programs, future studies should consider how differences in clinical training may influence clinician–AI collaboration and decisions.

Few studies to date have assessed how ML recommendations will be used by experts to support mental health care. Antidepressant selection in MDD represents a decision point where personalization of treatment offers the possibility of improving patient outcomes. This experimental study demonstrates how algorithmic errors and different types of explanations may influence clinicians’ treatment selection. Our results demonstrate that the implementation of ML tools with high accuracy rates may be insufficient to improve treatment selection accuracy, while also demonstrating the risk of overreliance when clinicians are shown incorrect treatment recommendations. These results demonstrate that evaluating ML models’ accuracy independently of their use by clinicians is not enough to determine real-world effectiveness. We do not argue that clinicians should be solely responsible for identifying algorithmic errors or biases. However, coping with imperfect algorithms will be a necessary step when such tools are used in the real world^46^. Our work helps to demonstrate how the design of these tools will influence this process.

## Supplementary information

Supplemental Material.

## Data Availability

The data that support the findings of this study are available from the corresponding author M.J. upon reasonable request.
