# Supplementary material for: How machine-learning recommendations influence clinician treatment selections: the example of antidepressant selection
Source: Transl Psychiatry. 2021 Feb 4;11:108. doi: 10.1038/s41398-021-01224-x (PMC7862671; doi:10.1038/s41398-021-01224-x)
Supplement: Supplementary file 1 — Supplemental Material. [file 41398_2021_1224_MOESM1_ESM.pdf]

Supplementary Material

S1: A complete list of patient vignettes and prescribing heuristics used in the heuristic-based explanations. For each question involving heuristic-based explanations, participants saw a list of heuristics that were relevant to the displayed patient vignette. Patients' names, age, and months of depressed mood were changed for each question.

Vignette #1: Sarah is a 34 year old woman who is single and works full time. She presents with 8 months of depressed mood. She has a history of arrhythmia, hypertensive heart disease, and diabetes. Current medications include amoxicillin. Prior treatment with Paroxetine did not cause a reduction in depression symptoms.

Vignette #1 Heuristics:

1. If concern for QT prolongation, favor Sertraline, avoid Citalopram
2. If avoiding weight gain, favor weight loss, favor Bupropion, avoid Mirtazapine
3. If concern for increased blood pressure, avoid SNRI's
4. If lack of response to Paroxetine, avoid SSRI's

Vignette #2: Sarah is a 34 year old woman who is single and works full time. She presents with 8 months of depressed mood and lack of appetite. She is underweight and has a history of obsessive-compulsive disorder. Current medications include celecoxib for osteoarthritis, and vitamin C. Prior treatment with Citalopram did not cause a reduction in depression symptoms.

Vignette #2 Heuristics:

1. If obsessive-compulsive, favor SSRI's, favor Clomipramine
2. If underweight of lack of appetite, favor weight gain, favor Mirtazapine
3. If underweight or lack of appetite, avoid appetite suppressants, avoid nausea-inducing, avoid SNRI's, avoid Sertraline
4. If lack of response to Citalopram, avoid SSRI's

Vignette #3: Sarah is a 34 year old woman who is single and works full time. She presents with 8 months of depressed mood. She is a moderate smoker and has a history of atrial fibrillation and atopic dermatitis. She is currently taking amoxicillin. Prior treatment with Paroxetine did not cause a reduction in depression symptoms.

Vignette #3 Heuristics:

1. If concern for QT prolongation, avoid SNRI's
2. If smoker, favor Bupropion
3. If lack of response to Paroxetine, avoid SSRI's

Vignette #4: Sarah is a 34 year old woman who is single and works full time. She presents with 8 months of depressed mood and fatigue. She has a history of diabetes and obesity. Prior treatment with Paroxetine did not cause a reduction in depression symptoms.

Vignette #4 Heuristics:

1. If obese, avoid weight gain, favor weight loss
2. If fatigue or lack of energy, favor activation, favor SNRI's
3. If lack of response to Paroxetine, avoid SSRI's

Vignette #5: Sarah is a 34 year old woman who is single and works full time. She presents with 8 months of depressed mood and lack of appetite. She has a history of seizure disorder, and current medications include Celecoxib and Omeprazole. Prior treatment with Citalopram did not cause a reduction in depression symptoms.

Vignette #5 Heuristics:

1. If underweight or lack of appetite, favor weight gain, favor Mirtazapine
2. If underweight or lack of appetite, avoid appetite suppressants, avoid nausea-inducing, avoid SNRI's, avoid Sertraline
3. If lack of response to Paroxetine, avoid SSRI's

S2: Final group sizes included in the analysis, accounting for skipped questions. We also removed from analysis any treatment selections not included in the 24 antidepressants scored by the psychopharmacologists.

Recommendation concordance	Explanation Type	Accuracy (N)	Confidence (N)	Perceived Utility (N)
Baseline	N/A	893	1096	N/A
Correct recommendations	Recommendation only	353	437	437
	Placebo	354	439	440
	Feature-based	348	440	439
	Heuristic-based	355	440	438
Incorrect recommendations	Recommendation only	178	220	220
	Placebo	169	220	219
	Feature-based	187	219	219
	Heuristic-based	176	220	219

S3: A summary of pairwise comparisons for the main effect of explanation type on treatment selection accuracy, for the baseline and incorrect recommendation conditions. P-values calculated using student's t-test. We include the raw p-values. Significance is denoted with a * and was determined using a Bonferroni correction of .05/10=.005.

Explanation 1	Explanation 2	t-statistic	p-value
Baseline	Feature-based	-3.57	.0004*
Heuristic-based	Feature-based	2.02	.044
Baseline	Recommendation only	-1.98	.048
Placebo	Feature-based	1.41	.159
Baseline	Placebo	-1.63	.103
Recommendation only	Feature-based	1.19	.233
Baseline	Heuristic-based	-0.90	.366
Heuristic-based	Recommendation only	-0.82	.411
Heuristic-based	Placebo	-0.58	.563
Placebo	Recommendation only	-0.23	.817
